# Ag/Au Alloyed Nanoislands for Wafer-Level Plasmonic Color Filter Arrays

**DOI:** 10.1038/s41598-019-45689-9

**Published:** 2019-06-24

**Authors:** Charles Soon Hong Hwang, Myeong-Su Ahn, Youngseop Lee, Taerin Chung, Ki-Hun Jeong

**Affiliations:** Department of Bio and Brain Engineering, KAIST Institute for Health Science and Technology (KIHST), Korea Advanced Institute of Science and Technology (KAIST), 291 Daehak-ro, Yuseong-gu, Daejeon, 34141 Korea

**Keywords:** Nanophotonics and plasmonics, Biomedical engineering

## Abstract

Alloyed metals in nanoscale exhibit some intriguing features that are absent in mono-metallic nanostructures. Here we report silver and gold alloyed nanoislands with high tunability of localized surface plasmon resonance (LSPR) wavelength in the visible range for wafer-level plasmonic color filter arrays. The nanofabrication includes two simple steps of concurrent thermal evaporation of Ag and Au grains and solid-state dewetting of the as-deposited nanocomposite thin film. The alloy ratio during the evaporation precisely tunes the LSPR wavelengths within 415–609 nm spectrum range. The elemental composition map reveals that alloyed nanoislands are completely miscible while preserving uniform size, regardless of the alloy ratio. Besides, the multiple lift-off processes and thermal dewetting of Ag/Au nanocomposite thin films successfully demonstrate the wafer-level nanofabrication of plasmonic color filter mosaic. Each plasmonic color pixel comprises different alloy ratio and efficiently transmits colors ranging from cyan, yellow, and magenta. The transmission spectra transposed onto a CIE 1931 color map show comparable color diversity to the plasmonic color filters fabricated by conventional e-beam lithographic techniques. This novel method provides a new direction for large-scale and visible plasmonic color filter arrays in advanced display or imaging applications.

## Introduction

Surface plasmons engage in extraordinary enhancements of electromagnetic fields near noble metal nanostructures^1^. In particular, tailoring of localized surface plasmon resonances (LSPR) in the optical region presents plasmonic materials as an ideal substrate for color filter applications^2–8^. Conventional color filters adopt chemically synthesized pigments as colorants that are naturally susceptible to several critical issues: performance degradation from extensive ultraviolet irradiation, highly accurate lithographic alignment for complex multilayered fabrication, and high crosstalk between neighboring pixels^9–11^. In contrast, plasmonic color filters provide enhanced material stability and color generation from ultrathin nanostructure arrays such as nanogratings^12,13^, nanoholes arrays^14,15^, nanodisks arrays^16^, nanoresonators^17^, *etc*. Such nanostructures are commonly accompanied with low-throughput top-down scanning-based nanolithographic fabrication processes including e-beam lithography and focus ion milling that are cost expensive and time consuming for wafer-level mass production. Attempts for large-area fabrication of plasmonic color filters has been actively demonstrated using interference lithography, colloidal self-assembly, or solid-state dewetting of thin films^18–20^. However, they still suffer from complex optical alignments, restrained geometric control, and insufficient enhancement or limited LSPR tuning.

Geometric dimensions and elemental compositions of metal nanostructure mainly determine the plasmon resonance wavelength^21,22^. Substantial works have demonstrated the precise tuning of LSPR wavelength with controllable geometric parameters such as size or period, yet the spectral tuning rarely covers the full visible range for color filter applications due to the intrinsic material property, i.e., the dielectric function^23,24^. For instance, aluminum, silver, and gold spherical nanostructures often cover the tuning range of 50 nm to 100 nm in the visible spectrum while copper, platinum, and tungsten are around 150 nm in the near infrared spectrum^25–30^. The broadband tuning of LSPR wavelength has been reported by controlling the shapes of nanostructures, but some technical issues such as polarization sensitivity and double plasmon resonance remain a concern. In contrast, controlling the elemental composition of metal nanostructures can provide many opportunities for tailoring the plasmon resonance^31^. Previous works on alloyed nanostructures have been mainly focused on solution-phase synthesis of noble metals. However, the alloy materials fabricated under solution-phase synthesis often suffer from particle aggregation, non-uniform distribution of nanoparticles upon substrate contact, and core-shell formation that causes double plasmon resonances or unstable plasmon resonance tailoring^32,33^. Recently, our group have demonstrated LSPR tuning of Ag/Au nanocomposites on micro/nano cellulose fibers and Ag/Au alloyed nanostructures from bi-layer thin films for label-free biosensing applications, but still have substantial technical limitations in serving as arrays of plasmonic color filters in broad band visible range^34–36^. Therefore, alloyed nanostructures for broad band tuning of LSPR wavelength with versatile nanofabrication still remain an unsolved task for plasmonic color filter array applications.

Here we report Ag/Au alloyed nanoislands for large-area plasmonic color filter arrays using solid-state dewetting of concurrently evaporated nanocomposite thin film. Noble metals Ag and Au are selected to feature LSPR wavelengths in the both far ends of the visible spectrum according to the dispersion relation. The CIE 1931 color space of Ag/Au alloyed nanoislands demonstrates highly diverse transmitted colors ranging from cyan, yellow and magenta, while tailoring the LSPR wavelength depending on the alloy ratio. In addition, this work successfully demonstrates plasmonic color filter arrays in the wafer-level using repeated lift-off processes, with each color pixel comprising Ag/Au nanoislands of different alloy ratios.

## Results and Discussion

**Figure 1:**
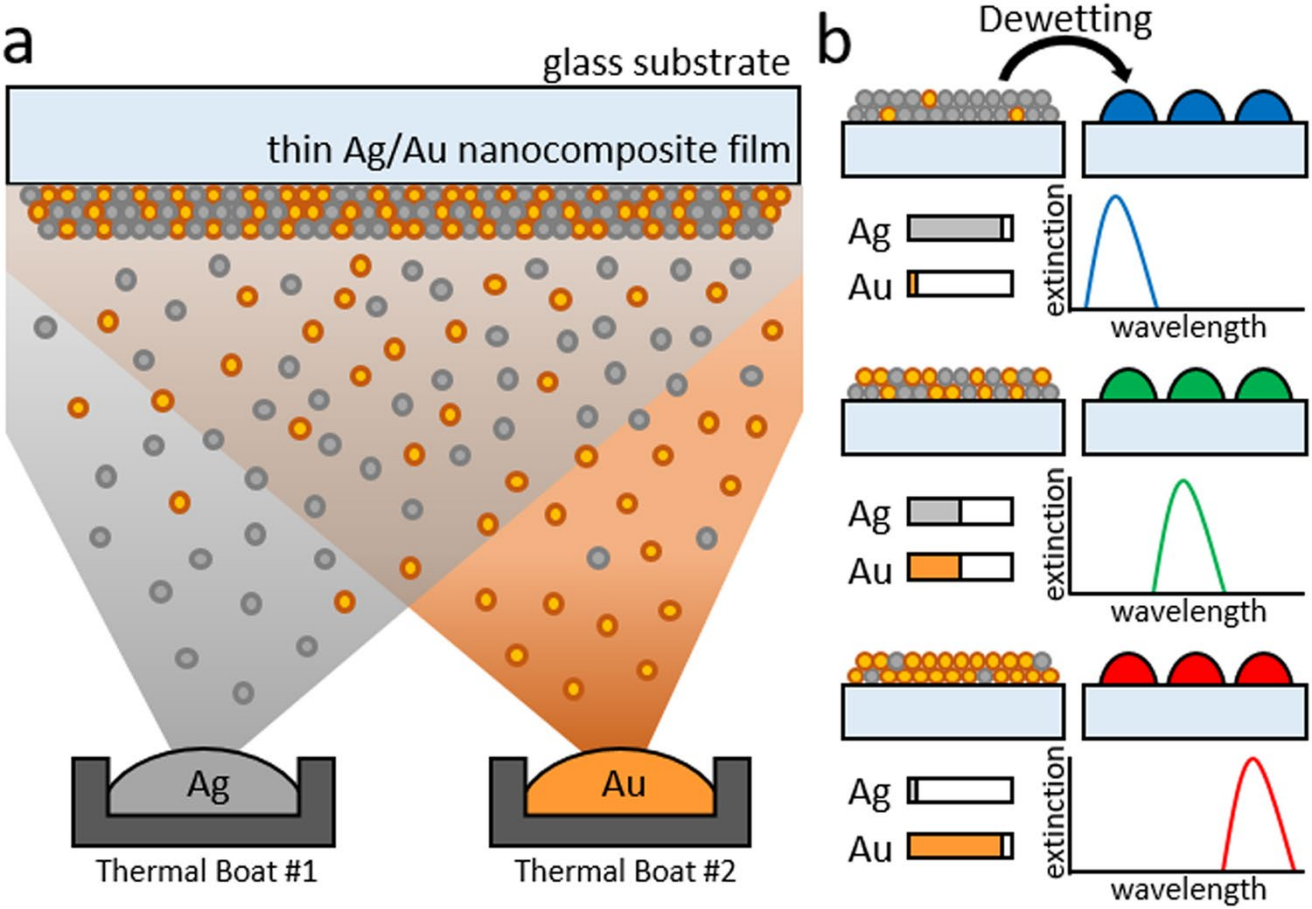
Large-area Ag/Au alloyed nanoislands for tuning localized surface plasmon resonance in a broadband visible spectrum. (**a**) The wafer-level nanofabrication method. An ultrathin Ag/Au nanocomposite film is concurrently evaporated on a substrate by using thermal evaporation. (**b**) The nanocomposite film is transformed into completely miscible Ag/Au alloyed nanoislands after solid-state dewetting. The metal evaporation rate simply controls the composition ratio of the nanocomposite, which determines the LSPR wavelength of the alloyed nanoislands in the broadband visible spectrum.

The wafer-level nanofabrication process for Ag/Au alloyed nanoislands is schematically illustrated in Fig. 1. A 10 nm thin Ag/Au nanocomposite film was first deposited onto a 4-inch quartz wafer substrate using concurrent thermal evaporation, during which both tungsten thermal boats were simultaneously heated. The evaporation rates of individual Ag and Au were typically set between 0.1 Å/s and 1.0 Å/s. Controlling the deposition rate of the individual boats via a crystal monitor enables the precise control of metal film’s composition ratio. The nanocomposite film was then thermal dewetted at 500 °C inside a box furnace to form Ag/Au alloyed nanoislands. The dewetting temperature was experimentally determined to fabricate highly circular nanoislands (Fig. S1). The plasmonic resonance of Ag/Au nanoislands is red-shifted for increasing Au alloy ratio, indicating wide spectral tailoring of LSPR wavelength by simple alteration of the alloy ratio as schematically shown in Fig. 1(b).

**Figure 2:**
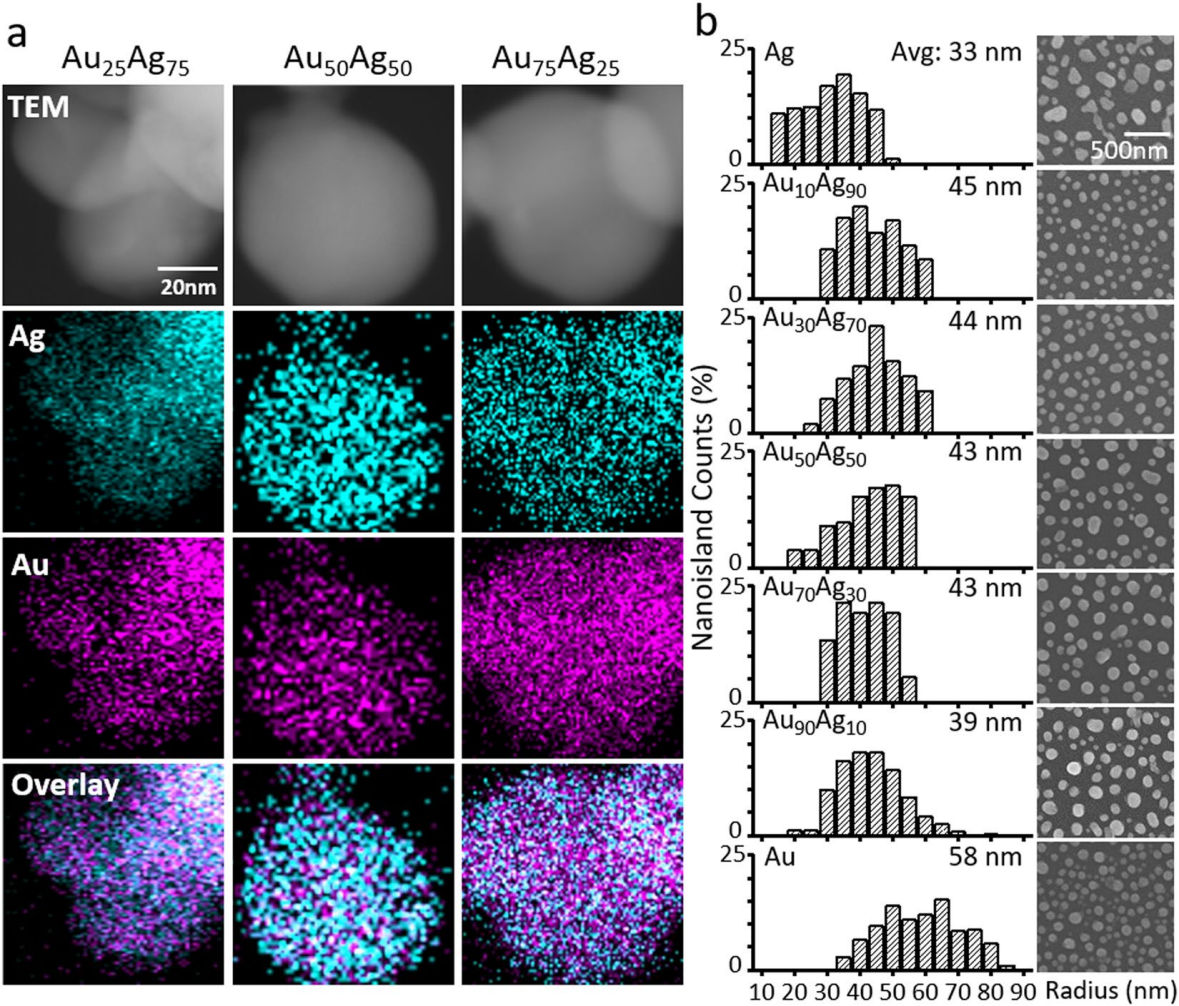
Miscibility and dimensional characterization of Ag/Au alloyed nanoislands. (**a**) EFTEM based EDS analysis of Ag/Au alloyed nanoislands depending on the alloy ratio. The measured alloy ratio of Ag/Au nanoislands (Au_24.9_Ag_75.1_, Au_44.4_Ag_55.6_, and Au_75.3_Ag_24.7_) matches well with the composition ratio of nanocomposite film (Au_25_Ag_75_, Au_50_Ag_50_, and Au_75_Ag_25_). The scattered distribution of the Ag and Au elements also confirms the complete miscibility of a single nanoisland. (**b**) Size distribution of alloyed nanoislands from SEM images. The average size of alloyed nanoislands is constant regardless of the alloy fraction.

The alloy miscibility and dimensional characterization of Ag/Au alloyed nanoislands of various alloy ratios are summarized in Fig. 2. Three samples with target alloy ratio of Au_25_Ag_75_, i.e., 25% Au and 75% Ag, Au_50_Ag_50_, and Au_75_Ag_25_ were fabricated for the elemental composition analysis using ultra-corrected-energy-filtered transmission electron microscope (UC-EF TEM) equipped with energy dispersive spectroscope (EDS) (Fig. 2a). Each four rows show TEM images, Ag/Au individual molecular distributions, and Ag/Au overlay distributions, respectively. The EDS elemental analysis confirms that Au_24.9_Ag_75.1_, Au_44.4_Ag_55.6_, and Au_75.3_Ag_24.7_ Ag/Au alloyed nanoislands were successfully fabricated for the targeted nanocomposite alloy ratio of Au_25_Ag_75_, Au_50_Ag_50_, and Au_75_Ag_25_, respectively. Moreover, the overlay EDS images reveal the scattered distribution of Ag and Au elements, verifying the complete miscibility of a single nanoisland. Next, eleven samples of nanoislands with alloy ratio of pure Au, Au_90_Ag_10_, Au_80_Ag_20_, Au_70_Ag_30_, Au_60_Ag_40_, Au_50_Ag_50_, Au_40_Ag_60_, Au_30_Ag_70_, Au_20_Ag_80_, Au_10_Ag_90_, and pure Ag were fabricated under the same fabrication conditions. Figure 2(b) shows the size distribution for the fabricated alloyed nanoislands for Au, Au_90_Ag_10_, Au_70_Ag_30_, Au_50_Ag_50_, Au_30_Ag_70_ Au_10_Ag_90_, and Ag samples. The scanning electron microscope (SEM) images indicate that the alloyed nanoislands exhibit highly uniform average radius of 43 nm regardless of the alloy ratio, while the pure Ag and Au nanoislands show slightly smaller and larger sizes of 33 and 58 nm, respectively. Figure S2 shows the size distribution of alloyed nanoislands for all eleven alloy ratios. This phenomenon is explicitly described by the difference in the material property during the solid-state dewetting. As-deposited Ag, Au, or Ag/Au nanocomposite metal films are poly-crystalline by nature. Since the dewetting process re-crystallizes Au into (111) dominant textures while Ag remains poly-crystalline, Au nanoislands show larger, angulated, and edged shapes compared to Ag nanoislands^36,37^. Note that even a minute addition of Ag to pure Au transforms nanoislands into spherical shapes because re-crystallization of Au is compensated by non-(111) textures of Ag, which provide higher chance of rim and boundary formation.

**Figure 3:**
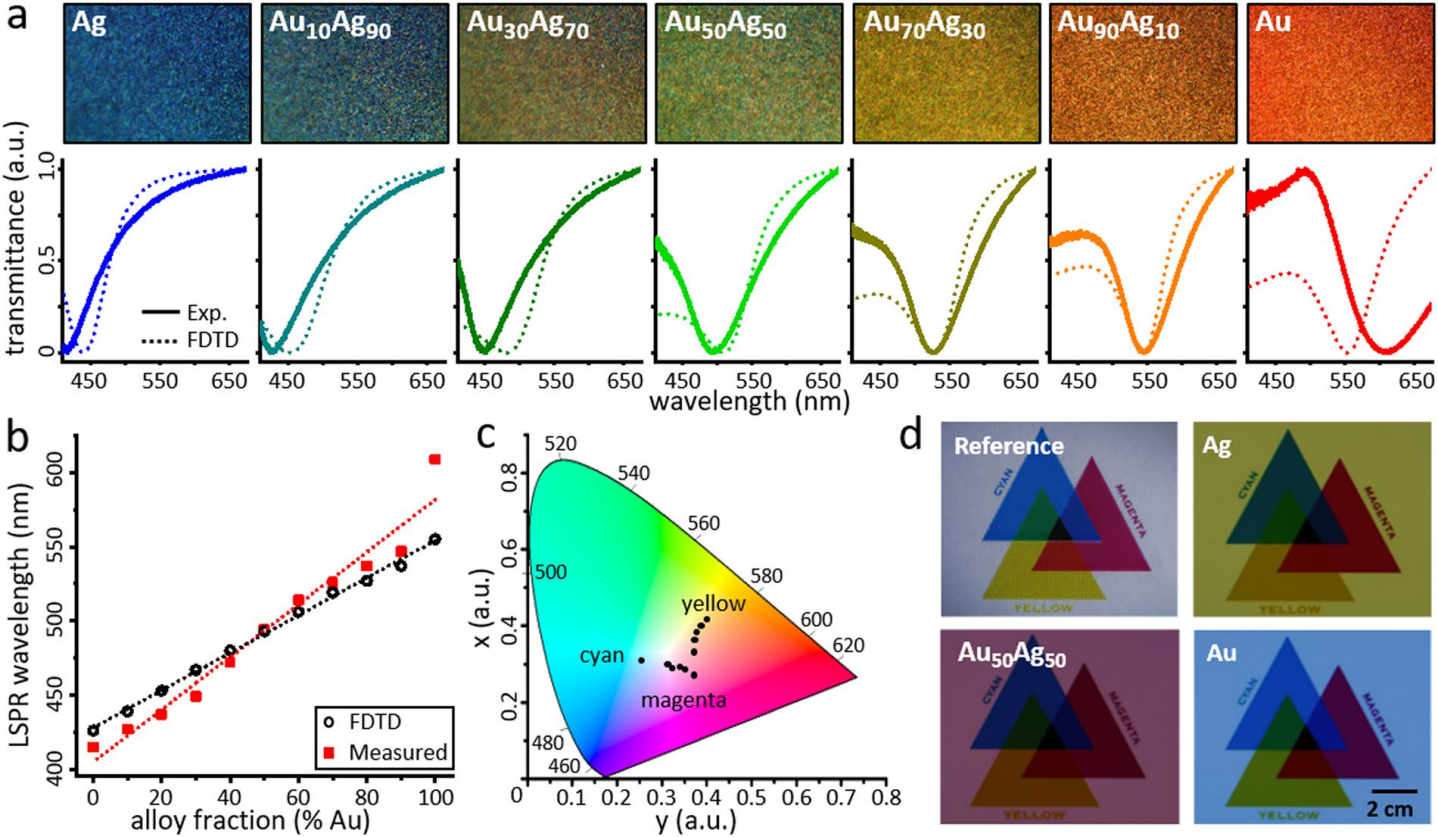
Plasmonic resonance of Ag/Au alloyed nanoislands. (**a**) Spectral transmittances of experimentally measured and FDTD calculated Ag/Au alloyed nanoislands compared with dark-field microscopic images depending on the alloy ratio. The measured resonance wavelength redshifts from 415 to 609 nm as the Au fraction increases, which matches well with the FDTD data shown in dotted line. The scattered color of the dark-field microscopic image is in accordance to the respective transmitted spectrums. (**b**) Comparison between the calculated and measured LSPR wavelengths. Both FDTD and measured values show linear relation of LSPR wavelength to the increase in alloy ratio. (**c**) Diagram of coordinates of CIE 1931 color space for Ag/Au alloyed nanoislands. Three vertexes of the measured transmittances are located in cyan, magenta, and yellow regions and thus various plasmonic colors can be created within the area of vertexes. (**d**) Filtered transmission image of a reference target for cyan, yellow, and magenta. Au, Au_50_Ag_50_, and Ag alloyed nanoislands substrates were placed in front of a commercial camera to obtain transmission images.

The tailoring of LSPR wavelengths in the visible spectrum with alloyed nanoislands provides ample selections of colors (Fig. 3). The sold lines in Fig. 3a show the normalized spectral transmittance of the fabricated alloyed nanoislands samples. The absolute transmission spectra of the same samples are illustrated in Fig. S3. The LSPR wavelength of nanoislands becomes red-shifted from 415, 427, 449, 494, 526, 547 to 609 nm, for increasing Au alloy ratio of Ag, Au_10_Ag_90_, Au_30_Ag_70_, Au_50_Ag_50_, Au_70_Ag_30_ Au_90_Ag_10_, and Au, respectively. This is in accordance to the back-scattered color images in the inset figure, captured using a dark-field mode microscope (Nikon, L-IM). The scattered colors encompass the myriad of colors ranging from dark blue, green, turquoise, yellow, and red. Note that LSPR wavelength is controlled only through adjusting the alloy ratio while other parameters (*i*.*e*., nanostructure size and surrounding environment) remain constant. The dotted lines Fig. 3a show the transmittance spectra for periodic array of Ag/Au alloyed nanoislands with varied mixing ratios calculated by three dimensional finite-difference time-domain (FDTD) method. The lumerical FDTD simulation of alloyed nanoislands were performed for periodic hemispherical nanostructure, of which the diameter and the period were obtained from SEM images of fabricated nanoislands in Fig. 2(b) using ImageJ software. The period was estimated assuming that the nanoislands were arranged in square array with uniform size. The material’s permittivity was calculated based on the composition-weighted equation ε_alloy_ = γ(n_Au_)^2^ + (1 − γ)(n_Ag_)2, where ε_alloy_ is the permittivity of the alloy, γ is the Au’s alloy ratio, and n_Au_/n_Ag_ are the refractive indice of Au and Ag, respectively^38–40^. The permittivity depending on the alloys ratio is summarized in Fig. S4. Figure 3b shows a direct comparison of the LSPR wavelengths of Ag/Au alloyed nanoislands between the experimental and the FDTD calculated results from Fig. 3a, both showing linear relation between the alloy ratio and the LSPR wavelengths. The dotted regression line for the experimental values shows a slightly higher slope than the FDTD values, caused by a noticeable deviation in pure Au nanoislands’ LSPR wavelength. The deviation found in pure Au nanoislands is due to the geometric mismatch between pure Au nanoislands and alloyed nanoislands, aforementioned in Fig. 2b. The LSPR wavelength becomes red-shifted for larger and angulated nanoparticles.

Figure 3c illustrates CIE 1931 color space calculated from the transmittance spectrum of various Ag/Au alloyed nanoislands, where x and y-axes represent combination of color, brightness, and intensity. Three vertices in the CIE color space are located in cyan, yellow, and magenta color regions, each corresponding Ag, Au_50_Ag_50_, and Au, respectively. All colors within the area of the three vertex can be further engineered by controlling the alloy ratio. The color diversity is comparable to recently published works on plasmonic color generation, yet this study successfully demonstrates large-area fabrication using concurrent evaporation and solid-state dewetting^3,41,42^. Moreover, recent works on noble metal alloys have demonstrated that alloying of Ag with Au significantly minimizes the surface oxidation of Ag thereby avoiding color distortion from silver corrosion^43,44^. Figure 3d shows transmitted color imaging using the fabricated Ag/Au alloyed nanoislands. The reference image was projected from a laptop screen, which was filtered through alloyed color filter substrates and imaged by a commercial DSLR camera (Canon, DS12619). Cyan, light magenta, dark magenta and yellow transmittance colors are observed for Au, Au_70_Ag_30_, Au_30_Ag_70_, and Ag substrates, respectively. Note that the transmittance color is complementary to the dark-field back-scattered colors in Fig. 3a insets.

The scattered and transmitted colors for Ag/Au alloyed nanoislands fabricated under different conditions are summarized in Fig. S5. The deposition thickness of Ag/Au nanocomposite film was controlled from 6, 9, 12, and 15 nm for Ag, Au_25_Ag_75_, Au_50_Ag_50_, and Au_75_Ag_25_ and Au alloy ratio samples were thermally dewetted at 300, 500, and 700 °C. Nanocomposite thin film dewetted at 300 °C shows scattered colors that were biased to the longer wavelengths, whereas 700 °C counterpart shows scattered colors biased to the shorter wavelengths due to average particle size difference caused by lack or excess of thermal energy. In contrast, dewetting at 500 °C successfully obtains colors ranging in the full visible band. In particular, Ag/Au alloyed nanoislands between 9 nm and 12 nm in initial deposition thickness display ample assortment of both scattered and transmitted colors.

**Figure 4:**
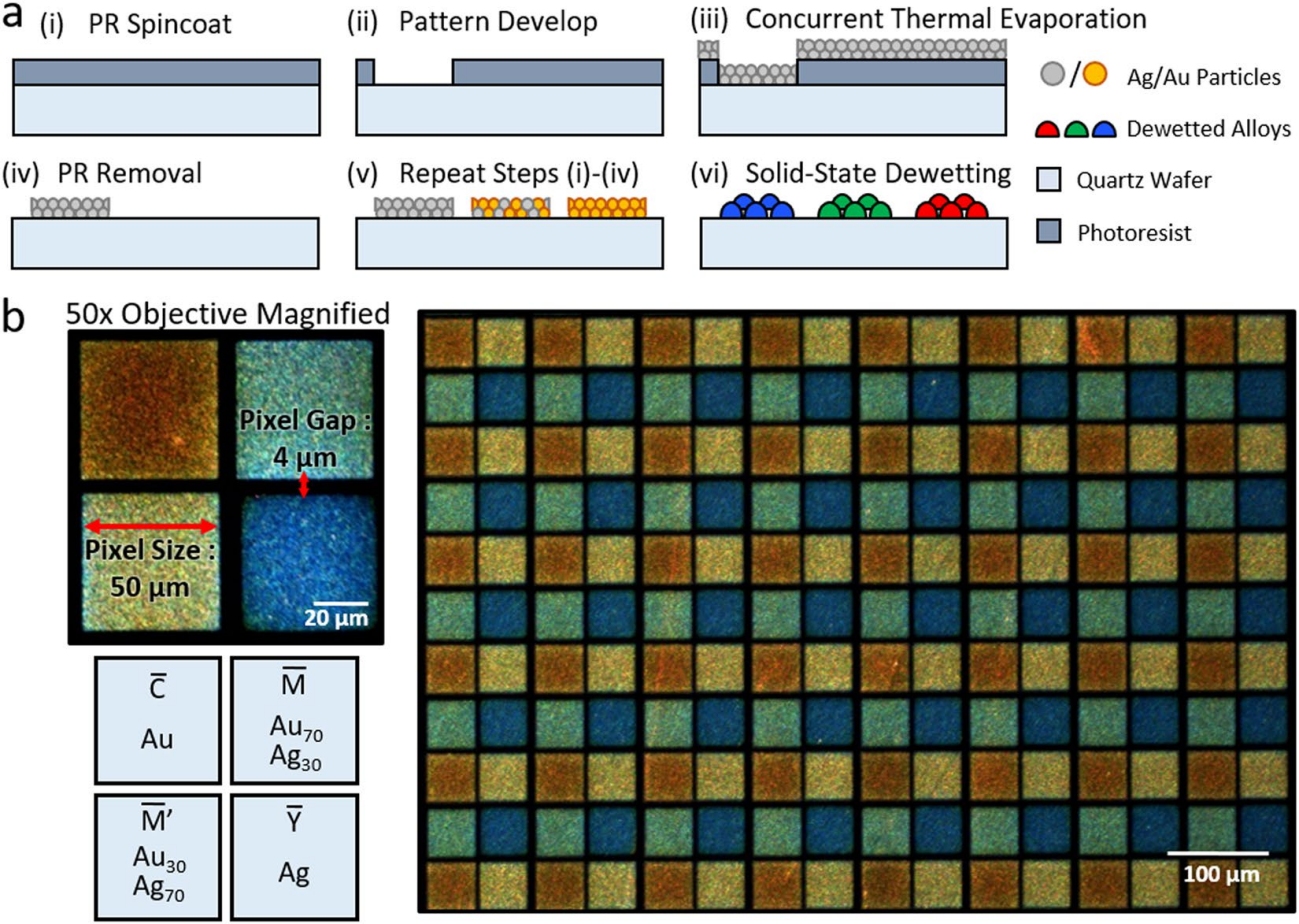
Plasmonic color filter mosaic using alloyed nanoislands. (**a**) Repeated lift-off process. Ultrathin nanocomposite films were sequentially and repeatedly evaporated, lifted-off, and thermally dewetted. (**b**) A dark-field microscopic image of plasmonic color mosaic with 50 × 50 µm^2^ pixel size, from 10 nm thick nanocomposite film of Au, Au_70_Ag_30_, Au_30_Ag_70_, and Ag. The RGB colors obtained from dark-field microscope are complementary to the scattered CYM scattered colors.

Wafer-level nanoalloyed plasmonic color filter mosaic was further fabricated by multiple lift off method onto a single quartz substrate (Fig. 4). First, 1.2 µm thick LOR and AZ1512 positive tone light-sensitive photoresist were deposited onto the substrate using spincoating. A pre-defined photomask and the substrate were aligned using commercial mask aligner (SUSS Microtec, MA6) and exposed under i-line UV (365 nm) light for 2.5 seconds at 16mJ/cm^2^. The photoresist was then developed with AZ MIF-300 and thin nanocomposite film was deposited by concurrent thermal evaporation. Next, the developed photoresists were stripped with MR-REM-700. The above processes were repeated four cycles by manually aligning and shifting the chrome mask, while depositing Au, Au_70_Ag_30_, Au_30_Ag_70_, and Ag nanocomposite film during each cycle. Finally, the substrate was thermally dewetted inside box furnace for 60 minutes at 500 °C to fabricate wafer-level plasmonic color filter arrays. The dark-field microscopic image of the fabricated color filter arrays is presented in Fig. 4b. A single color pixel has 50 × 50 µm^2^ dimension, with 4 µm inter-pixel distance. The dark-field colors of each color filter pixels provides red, yellow, green and blue colors depending on the alloy ratio that are complementary to CMY color systems.

To conclude, this work successfully demonstrated Ag/Au alloyed nanoislands for large-area plasmonic color filter arrays using concurrent thermal evaporation and solid-state dewetting. The LSPR wavelengths of the alloyed substrates is effectively controlled from 415 to 609 nm range by adjusting the Au ratio, which in response generates scattered RGB mode or transmitted CYM color sets. Wafer-level alloyed plasmonic color filter arrays of 50 µm × 50 µm^2^ dimension has also been fabricated using repeated lift-off processes. The alloyed plasmonic color filter exhibits comparable color diversity in comparison to the conventional e-beam lithography based plasmonic color filters. This novel wafer-level plasmonic arrays of alloyed nanoislands provide new outlooks for advanced display or imaging applications.

## Methods

### Solid-state dewetting of thin films

The as-deposited Ag/Au nanocomposite thin film on glass substrate was placed inside a box furnace. The temperature was steadily increased to 500 °C from the room temperature and kept constant for one hour. The ramp-up and ramp-down temperature rates of the box furnace were 20 °C/s and 5 °C/s, respectively, which were chosen to minimize the thermal stress applied to the samples.

### Transmittance spectrum measurements

The transmittance spectra of Ag/Au alloyed nanoislands were measured with an inverted microscope (Carl Zeiss, Axiovert 200M) equipped with a commercial spectrometer (Princeton Instruments, MicroSpec 2300i) and a charge-coupled device camera (Princeton Instruments, PIXIS: 400BR). The transmittance spectra were collected with 50x objective lens.

### Dark-field microscopy

The back-scattering colors of the Ag/Au alloyed nanoislands were observed with dark-field mode of microscope (Nikon, L-IM), equipped with illumination module (Nikon, LV-UEPI2) and halogen lamp (Olympus, TH4-200), and captured using charge-coupled device camera (Teledyne Photometrics CoolSNAP-cf).

## Supplementary information

Additional information on the geometric and optical characterization of Ag/Au alloyed nanoislands are summarized in the Supporting Information.
