# Supplementary material for: Ag/Au Alloyed Nanoislands for Wafer-Level Plasmonic Color Filter Arrays
Source: Sci Rep. 2019 Jun 24;9:9082. doi: 10.1038/s41598-019-45689-9 (PMC6591299; doi:10.1038/s41598-019-45689-9)
Supplement: Supplementary file 1 — Additional information on the geometric and optical characterization of Ag/Au alloyed nanoislands are summarized in the Supporting Information. [file 41598_2019_45689_MOESM1_ESM.docx]

Supporting Information

**Title** Ag/Au Alloyed Nanoislands for Wafer-Level Plasmonic Color Filter Arrays

Charles Soon Hong Hwang, Myeong-Su Ahn, Youngseop Lee, Taerin Chung, and Ki-Hun Jeong*


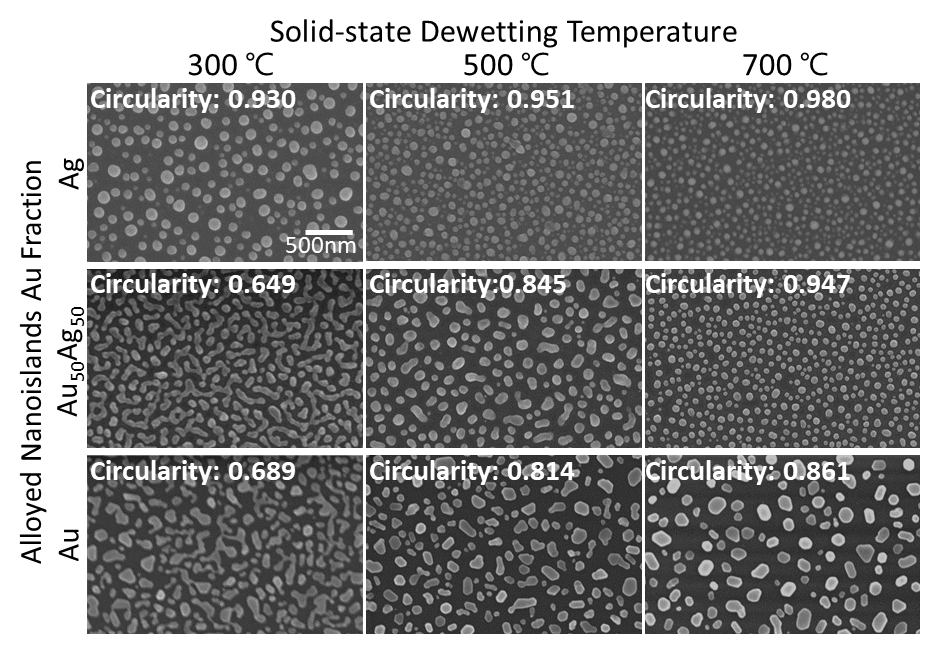


Figure S1. The average circularity of Ag/Au alloyed nanoislands depending on the temperature during solid-state dewetting. The average circularity was calculated by the ratio of the area to the square of the perimeter, ranging from 0 to 1. Higher circularity index indicates a more circular shape. Alloyed nanoislands fabricated at 300℃ shows relatively lower circularity index due to insufficient thermal energy. In contrast, dewetting at higher temperatures, i. e. 500 ℃ or 700 ℃ enables the fabrication of circular nanoislands with index exceeding 0.8. However, thermal dewetting at 700 ℃ results in re-evaporation of Ag metals to neighboring sites, which needs to be avoided for the successful fabrication of high performance wafer-level plasmonic color filter arrays.


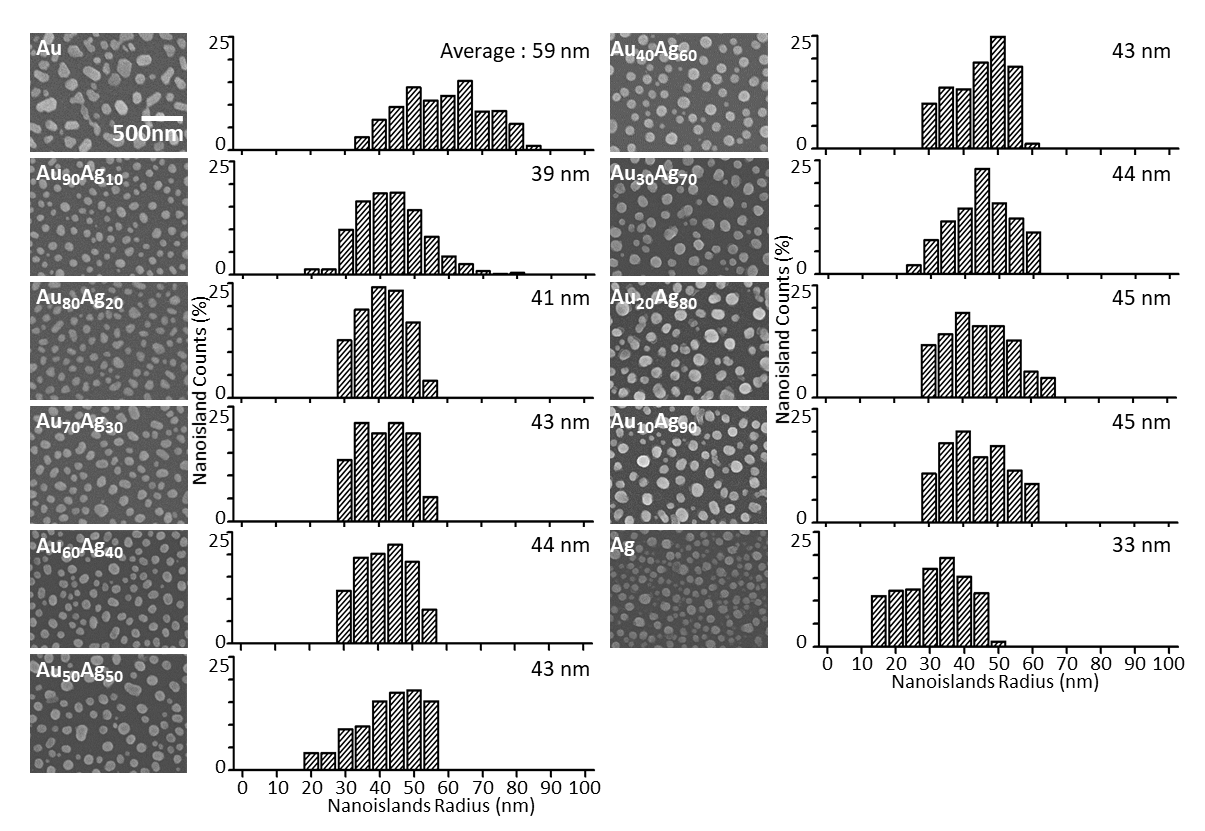


Figure S2. Scanning electron microscopic images and the size distribution of Ag/Au alloyed nanoislands depending on the alloy ratio. The eleven samples were fabricated under the same conditions: 10 nm film deposition, thermal dewetting at 500 ℃ for 1 hour, 20 °C/s ramp up temperature, and 5 °C/s ramp down temperature. The effective radius of alloyed nanoislands was calculated using ImageJ software from the SEM images. The size distribution indicates that alloyed nanoislands sustain a comparably uniform size after thermal dewetting.


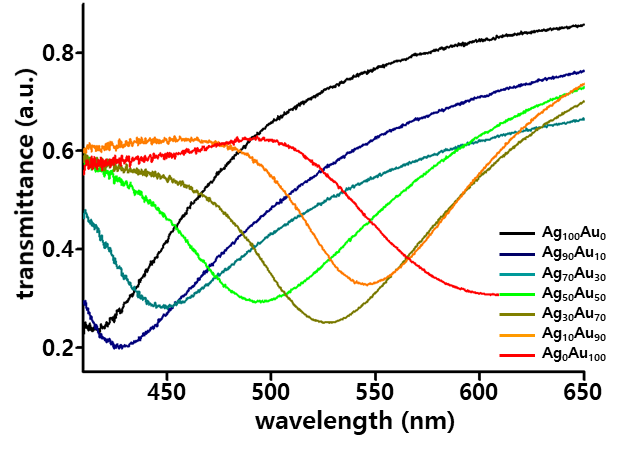


Figure S3. Transmittance spectra of Ag/Au alloyed nanoislands with Au ratio of 0, 10, 30, 50, 70, 90, and 100%. The absolute transmittance at the resonance wavelengths of the Ag/Au nanoislands slightly increases for increasing alloy ratio of Au due to the permittivity index difference between Ag and Au. The real and imaginary part of the dielectric function are respectively related with the strength and the loss in the plasmonic materials. Since Ag/Au alloys with higher Au composition has higher imaginary permittivity as shown in Figure S2, we observe higher ohmic loss.


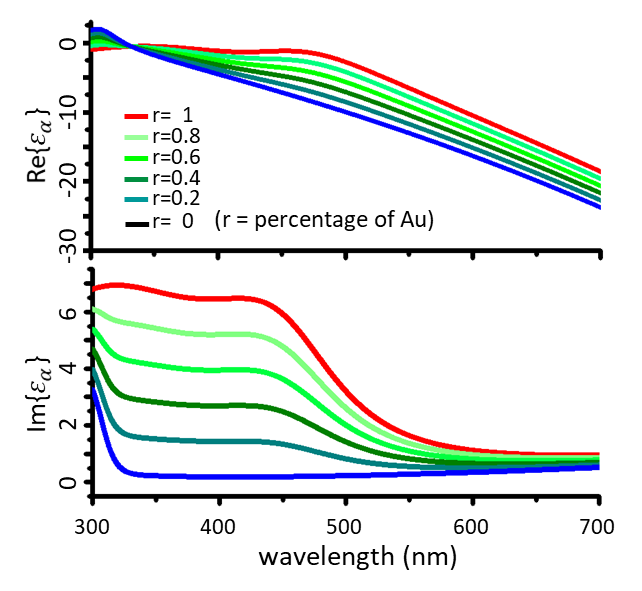


Figure S4. Calculated real and imaginary permittivity index for Ag/Au alloyed nanostructures. Permittivity for alloyed nanostructures with 0, 20, 40, 60, 80 and 100% Au ratio was numerically calculated using the composition-weighted equation. Johnson/Christy and Palik (0-2um) material library were used for gold and silver, respectively, during the calculation.


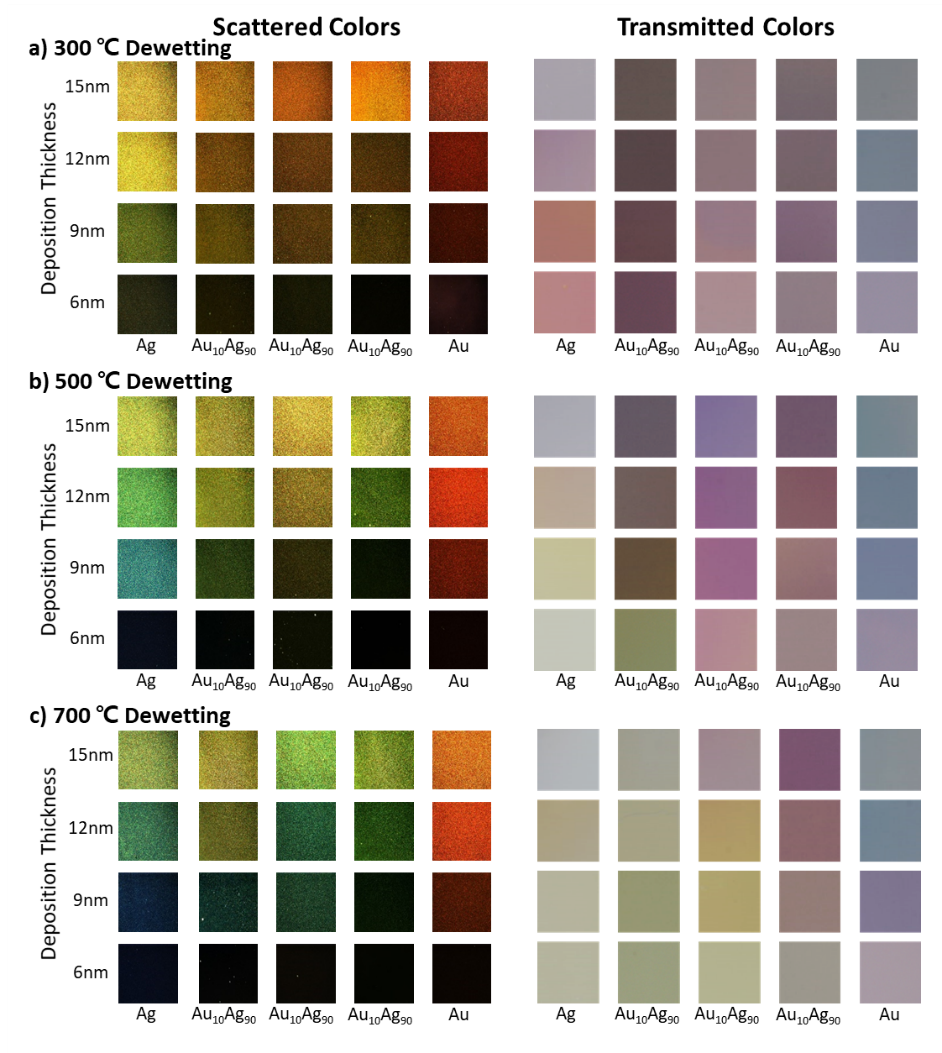


Figure S5. Dark-field scattered colors and transmitted colors for Ag/Au alloyed nanoislands depending on the deposition thickness and alloy ratio for a) 300 °C b) 500 °C and c) 700 °C thermal dewetting temperatures. Alloyed nanoislands fabricated under insufficient temperature may cause undesirable rim boundaries, which result in angulated or mesh-type nanostructures and red-shift the LSPR wavelength. In contrast, thermal dewetting with excessive heat may also form smaller alloyed nanoislands that blue-shift the LSPR wavelengths. Therefore, dewetting at 300 °C generally lacks in blue for scattered colors and yellow in transmitted colors, while dewetting at 700 °C lacks in red for scattered colors and cyan for transmitted colors. Alloyed nanoislands dewetted at 500 °C show a good balance of RGB and CMY colors under different fabrication conditions. In particular, Ag/Au alloyed nanoislands between 9nm and 12nm in initial deposition thickness display ample assortment of both scattered and transmitted colors.
